# Size-dependent endocytosis of gold nanoparticles studied by three-dimensional mapping of plasmonic scattering images

**DOI:** 10.1186/1477-3155-8-33

**Published:** 2010-12-20

**Authors:** Sheng-Hann Wang, Chia-Wei Lee, Arthur Chiou, Pei-Kuen Wei

**Affiliations:** 1 Research Center for Applied Sciences, Academia Sinica, 128, section 2, Academia Road, Nankang, Taipei 11529, Taiwan; 2 Institute of Biophotonics Engineering, National Yang Ming University, Taipei, 112, Taiwan; 3 Department of Material Science and Engineering, National Taiwan University, 1, Section 4, Roosevelt Road, Taipei, Taiwan 10617

## Abstract

**Background:**

Understanding the endocytosis process of gold nanoparticles (AuNPs) is important for the drug delivery and photodynamic therapy applications. The endocytosis in living cells is usually studied by fluorescent microscopy. The fluorescent labeling suffers from photobleaching. Besides, quantitative estimation of the cellular uptake is not easy. In this paper, the size-dependent endocytosis of AuNPs was investigated by using plasmonic scattering images without any labeling.

**Results:**

The scattering images of AuNPs and the vesicles were mapped by using an optical sectioning microscopy with dark-field illumination. AuNPs have large optical scatterings at 550-600 nm wavelengths due to localized surface plasmon resonances. Using an enhanced contrast between yellow and blue CCD images, AuNPs can be well distinguished from cellular organelles. The tracking of AuNPs coated with aptamers for surface mucin glycoprotein shows that AuNPs attached to extracellular matrix and moved towards center of the cell. Most 75-nm-AuNPs moved to the top of cells, while many 45-nm-AuNPs entered cells through endocytosis and accumulated in endocytic vesicles. The amounts of cellular uptake decreased with the increase of particle size.

**Conclusions:**

We quantitatively studied the endocytosis of AuNPs with different sizes in various cancer cells. The plasmonic scattering images confirm the size-dependent endocytosis of AuNPs. The 45-nm-AuNP is better for drug delivery due to its higher uptake rate. On the other hand, large AuNPs are immobilized on the cell membrane. They can be used to reconstruct the cell morphology.

## Background

Gold nanoparticles (AuNPs) are important nanomaterials in biomedicine where they can be used to achieve drug delivery and photodynamic therapy [1-6]. For biomedical applications, a thorough understanding of the mechanisms of AuNP cellular entry and exit is required. In previous studies, the endocytosis of AuNPs was found to be not only dependent on the surface coating but also on particle size [7-12]. In these studies, AuNPs were observed by using electron microscopy or fluorescent optical microscopy. Several drawbacks are inherent in these methods, since cells are not alive when they are observed by electron microscopy, and fluorescent labelling suffers from problems with photobleaching. Long-term observation is not attainable by the fluorescent technique. Additionally, quantitative estimation of AuNP numbers in cells is not easy using fluorescent signals. In this paper, we present a label-free method for long-term tracking of the movement of AuNPs with different sizes. A three-dimensional (3D) image process was developed to identify the distribution of AuNPs. Using the 3D distribution, the uptake efficiencies for different sizes of AuNPs were compared.

The label-free method was based on the large difference between the scattering spectra of AuNPs and cellular organelles. AuNPs are known to have broad optical absorption/scattering for visible and near-infrared light due to the excitation of localised surface plasmon resonance (LSPR). The scattering cross-section of a nanoparticle is usually described by the Mie scattering theory [13,14].

(1) $$C_{s}(\lambda )={\frac{32\pi }{3\lambda^{4}}}^{4}r^{4}n^{4}\frac{{\left[\varepsilon_{r}(\lambda )-n^{2}\right]}^{2}+\varepsilon_{i}^{2}(\lambda )}{{\left[\varepsilon_{r}(\lambda )+2n^{2}\right]}^{2}+\varepsilon_{i}^{2}(\lambda )}$$

**Figure 1:**
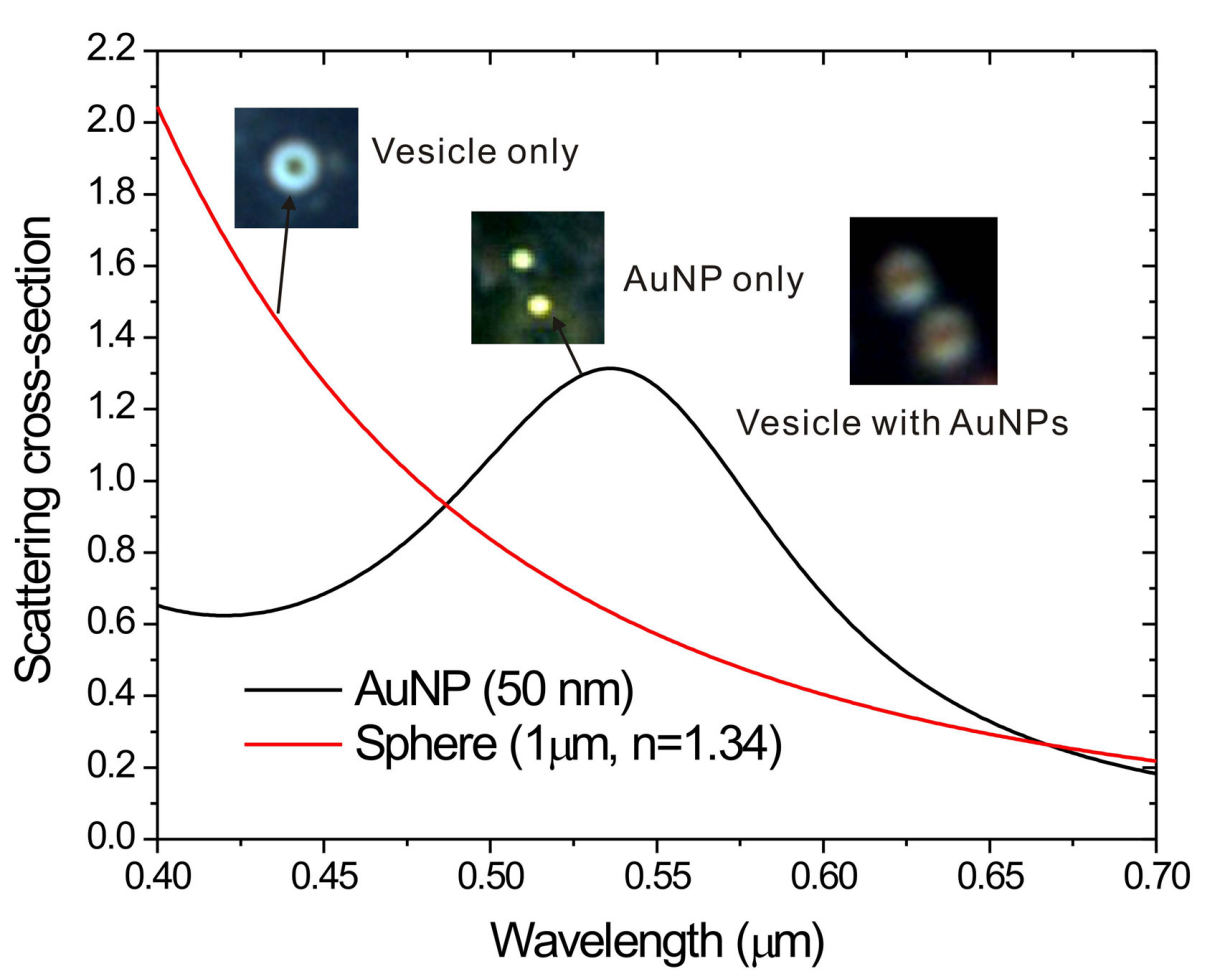
**The scattering spectra for different nanoparticles**. The particles were 50-nm-AUNP and a 1-μm dielectric sphere in water medium. The inset shows the measured scattering images for single vesicle, AuNP and vesicle containing AuNPs. The nanometer-size AuNP has a comparable scattering intensity with microspheres. However, the AuNP shows yellow color and the vesicle shows blue. When endocytosis of AuNPs happens, the AuNPs became orange with blue surroundings. The optical scattering images can be used to identify the uptake of AuNPs.

Where *r *is the radius of the nanoparticle, *λ *is the incident wavelength, *n *is the refractive index of environmental medium and *ε*_*r *_and *ε*_*i *_are the real and imaginary parts of the dielectric constant of the nanoparticle, respectively. The AuNP has a negative dielectric constant. Large scattering occurs when *ε*_*r *_(*λ*) = -2*n*^2^. In an aqueous environment (*n *= 1.332), the wavelength for maximum scattering is about 550-600 nm. On the other hand, the dielectric constant of cellular organelles is positive. The scattering efficiency is proportional to ${\left(\frac{1}{\lambda }\right)}^{4}$. The shorter wavelength has a larger scattering. The large spectral difference makes different colours for AuNPs and celluar organelles. For example, Figure 1 shows the calculated spectra for a 50 nm AuNP and a 1 μm diameter dielectric sphere (*ε*_*r *_= 1.34^2^) in an aqueous medium. The nanometre AuNP has a comparable scattering intensity with the micrometre sphere, but the single 50 nm AuNP shows as yellow and the dielectric sphere shows as blue. When endocytosis of AuNPs occurs, the AuNPs are surrounded with a dielectric coating. The scattering image is visualised as an orange centre with a blue periphery. The insets of Figure 1 are the measured scattering images for a 45 nm AuNP and a micrometre vesicle. The AuNPs in vesicles can be directly identified by the coloured scattering images. Such spectral differences can be used to distinguish AuNPs from cellular organelles. In this paper, we proposed an image processing method to identify the 3D distribution of AuNPs in living cells. The endocytosis of AuNPs with different sizes, including 45 nm, 70 nm and 110 nm, were compared.

## Materials and methods

### Dark-field optical sectioning microscopy

**Figure 2:**
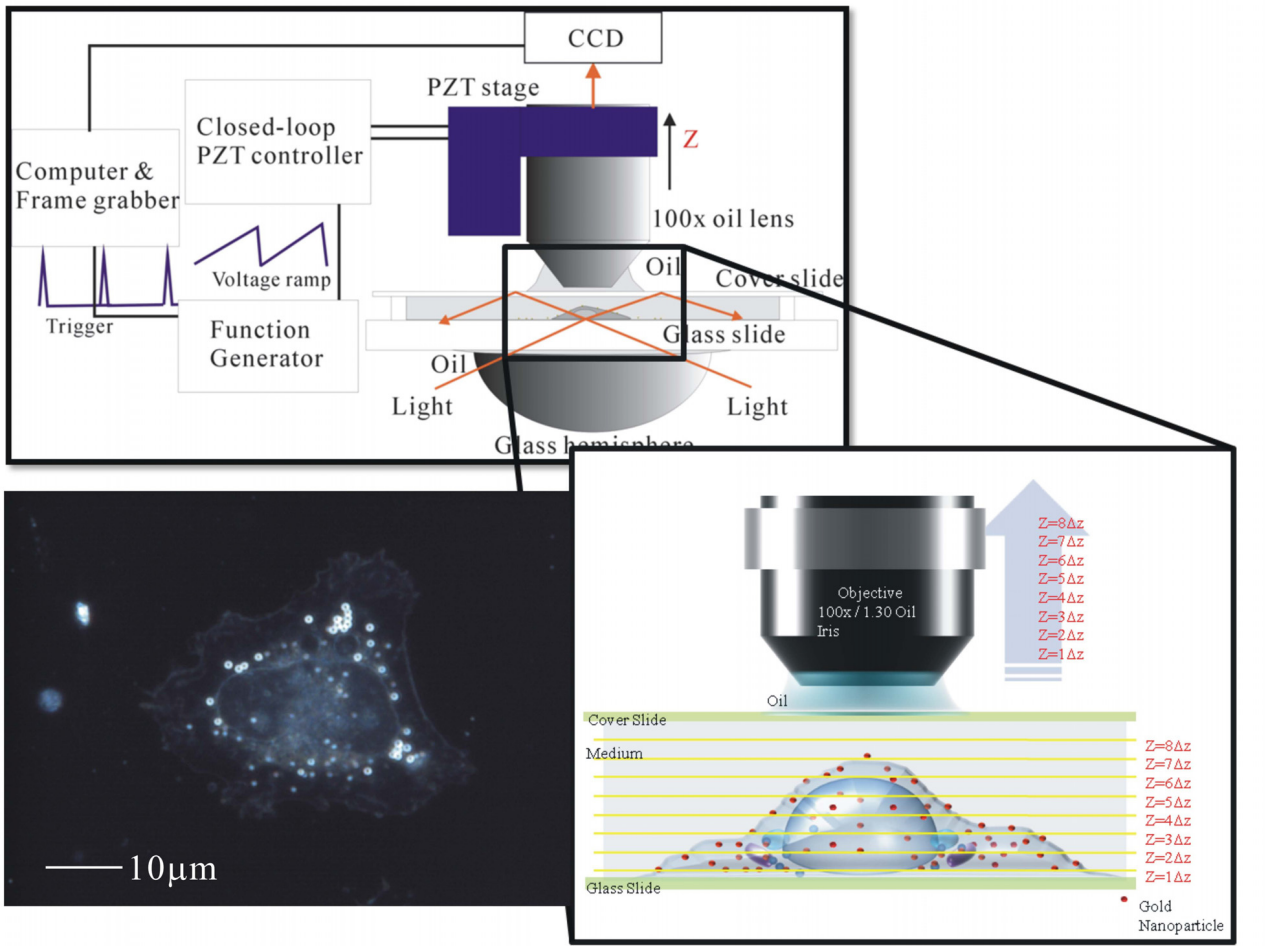
**The optical setup of a dark-field optical section microscope**. The light source was a 60 W metal halide light. It illuminated the samples through a hemisphere glass lens. The illumination light had a large incident angle in the medium, only the scattering light was collected by the objective lens and imaged by a color CCD. We recorded the scattering images at different focal positions by using a quickly linear scan of the objective lens along the depth direction. The left image is a dark-field CCD image for a HeLa cell without any AuNPs.

The 3D scattering images of AuNPs and cellular organelles were mapped by using a dark-field optical sectioning microscope [15]. For long-term observation of the cells, the microscopic system was put in a chamber to maintain a 37°C humidified atmosphere. Figure 2 illustrates the optical setup. The light source was a 60 W metal halide lamp which illuminated the samples through a hemispheric glass lens. The incident angle was larger than the critical angle between glass and air. Therefore, only scattering light could be collected by the objective lens. The lens (100×, NA = 1.35) was mounted on an objective piezo nano-focusing system. For 3D images, the scattering images at different focal planes were taken by a fast scan of the objective along the depth direction. The nano-focusing system was controlled by a function generator which generated a voltage ramp to move the objective. A trigger signal was simultaneously sent to the frame grabber to begin recording a sequence of CCD images. In dark-field sectioning microscopy, the voltage from the controller was 20 V. It made a 16 μm movement from the focal position. The scan frequency was 0.5 Hz. The CCD acquisition time was 50 ms, yielding 40 images during a scan.

### Cells and incubation

In the experiments, we studied the interactions of AuNPs with two kinds of cancer cells, non-small lung cancer cells (CL1-0) and HeLa cells. Both cells have lateral dimensions of about 20 μm and heights about 8 μm. These cells were cultured on cleaned glass slides with thin square chambers to hold the medium. They were maintained in RPMI medium (GIBCO) supplemented with 10% FBS (fetal bovine serum) (GIBCO) at 37°C in a humidified atmosphere. The cells were cultured for 24 hours to ensure that they adhered well onto the glass slides. The left image in Figure 2 shows a dark-field CCD image for a HeLa cell without any AuNPs. This image indicates that no colorful spots are in the cell. The ring patterns are the micrometre vesicles.

### Fabrication of AuNPs

AuNPs were prepared by using the reduction of chloroauric acid (H[AuCl_4_]) solution [16,17]. Different diameters of AuNPs were made by using different ratios of chloroauric acid and reducing agent. The fabrication parameters for different sizes of AuNPs were listed in Table 1. We used the SEM to determine the mean AuNP size and its size dispersion. Additional file 1 shows the SEM images for 13 nm, 45 nm, 70 nm and 110 nm AuNPs on glass substrates. We used the measurement function in the SEM (LEO-1530) to measure the diameter for each nanoparticle. About 100 nanoparticles were measured for each size. The mean size and size dispersion of the AuNP are listed in Table 2.

**Table 1:** The parameters for making different sizes of gold nanoparticles

Gold nanoparticle size (nm)	**HAuCl**_**4**_	Reducing Agent	Temperature
13	50 ml, 10 mM	5 ml, 38.8 mM Sodium Citrate	130°C

45	50 ml, 0.3 mM	0.5 ml, 38.8 mM Sodium Citrate	130°C

70	50 ml, 0.3 mM	0.4 ml, 38.8 mM Sodium Citrate	130°C

110	0.75 ml, 25 mM	330 μl, 37% Formaldehyde	Room Temperature

**Table 2:** The zeta potentials on the surface of gold nanoparticles before and after the surface modification of ssDNA. The ssDNAs carry negative charges that make the surface potentials more negative after the modification.

Size of gold nanoparticles (nm)	Zeta potential before ssDNA conjugated (mV)	Zeta potential after ssDNA conjugated (mV)
13 ± 2.6	-13.99 ± 1.75	-27.27 ± 1.03

45 ± 3.1	-17.83 ± 1.31	-28.69 ± 1.07

70 ± 4.9	-19.14 ± 1.48	-24.66 ± 1.88

110 ± 5.1	-10.25 ± 0.80	-19.48 ± 0.97

### Surface modification of AuNPs

The surface modification is important for the endocytosis of AuNPs. For AuNPs without ligands, they cannot interact with cells. The unmodified AuNPs will be on the glass substrate [18]. To make AuNPs interacting with cancer cells, we modified the AuNP surface with single-stranded DNA (ssDNA) sequences. The DNA sequence was SH-(CH_2_)_10_-GCAGTTGATCCTTTGGATACCCTGG, where the thiol group enabled covalent bonding between the ssDNA and gold surface. This ssDNA segment was an aptamer for cellular surface mucin glycoprotein (MUC1) which is over-expressed in the extracellular matrix of cancer cells [19-22]. It is noted that in the preparation of AuNPs, the sodium citrate acted as a reducing agent. The negatively-charged citrate ions were adsorbed on the gold nanoparticles, introducing negative surface charges. It is known that DNAs also carry negative charges. If DNA aptamers were immobilised on the AuNP surface, it made the surface charge more negative. We used a zeta-potential analyzer (Brookhaven 90Plus) to measure the surface potential. The electrostatic potential on the particle surface is called the zeta potential. In the measurement, we applied unit field strength (1 Volt per metre) to the AuNP solution. The electrophoretic mobility of AuNPs was measured based on dynamic light scattering. There are theories that link electrophoretic mobility with zeta potential. The calculated zeta potentials for different size of AuNPs are listed in Table 2. It can be seen that after the interaction with DNA aptamers, the AuNPs increased negative surface charges. It confirmed that the DNA aptamers were immobilised on the AuNP surface.

### Preparation of AuNP aggregates in submicron holes

When endocytosis of AuNPs occurs, the AuNPs are wrapped by the vesicles. The vesicle size are most in submicron scale and the AuNPs in the vesicle are in aggregated form. To find the relation between the scattering optical intensity and number of AuNPs in the vesicle. We prepared 500-nm-diameter holes in a transparent film to mimic the vesicles. The transparent film was coated on a glass substrate. The glass surface was modified with 4-mercaptobenzoic acid, sodium borohydride, hydrogen peroxide (27.5 wt% solution in water) and 3-aminopropyltriethoxysilane (APTES) in order to immobilize the AuNPs [23]. The sample was dipped in the AuNP solution. After six hours of interaction time, we washed the sample and measured the scattering images in water. The measured sample was then dried and observed by the SEM to identify the number of AuNPs in each hole.

## Results

### Cell-nanoparticle interactions

**Figure 3:**
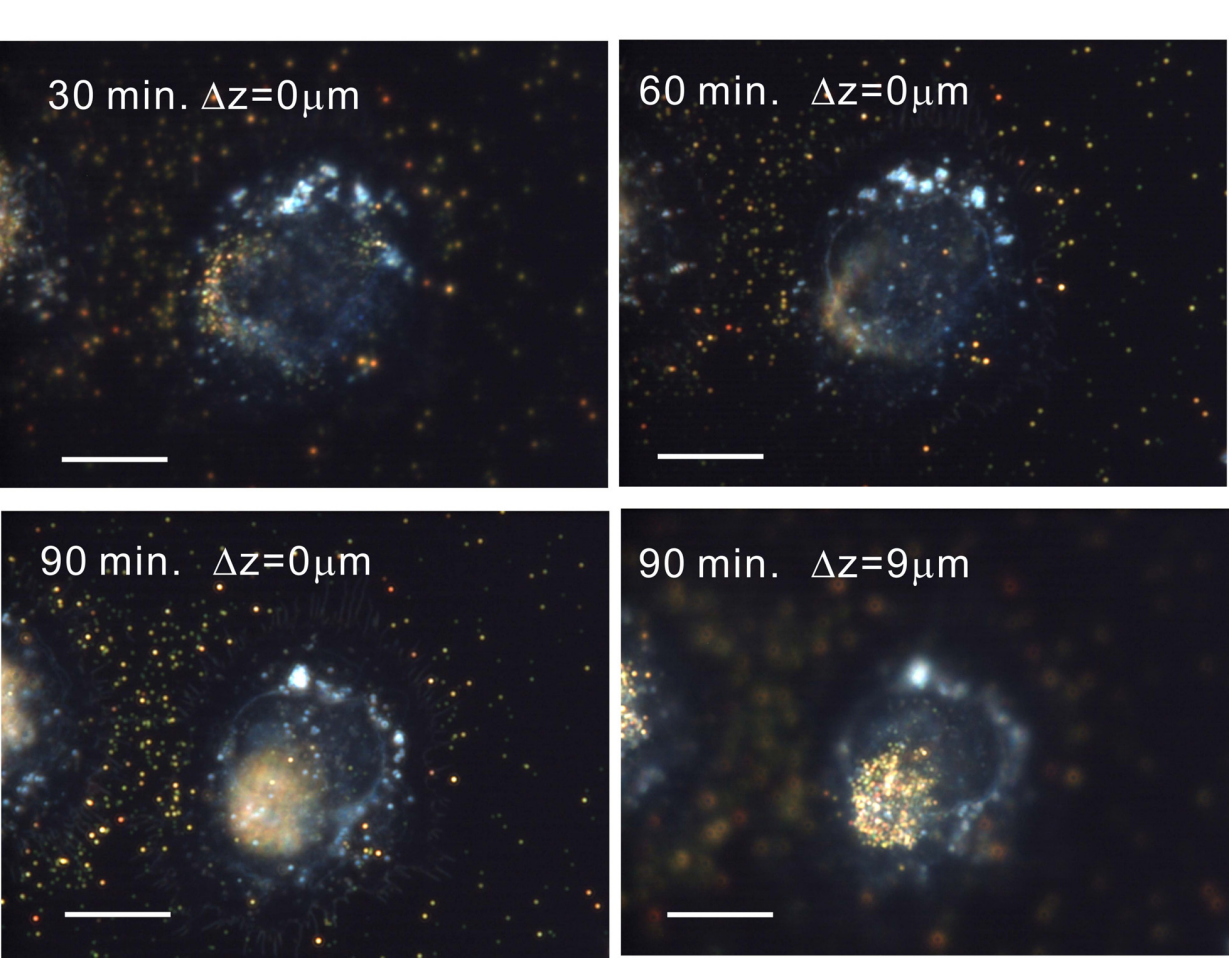
**The dark-field images for 70-nm-AuNPs and cells**. The images show 70-nm-AuNPs and HeLa cells at different interaction time. The last image was taken at top of the cell. It shows that many AuNPs were sent to top of the cell membrane without being internalized. The scale bar is 10 μm.

**Figure 4:**
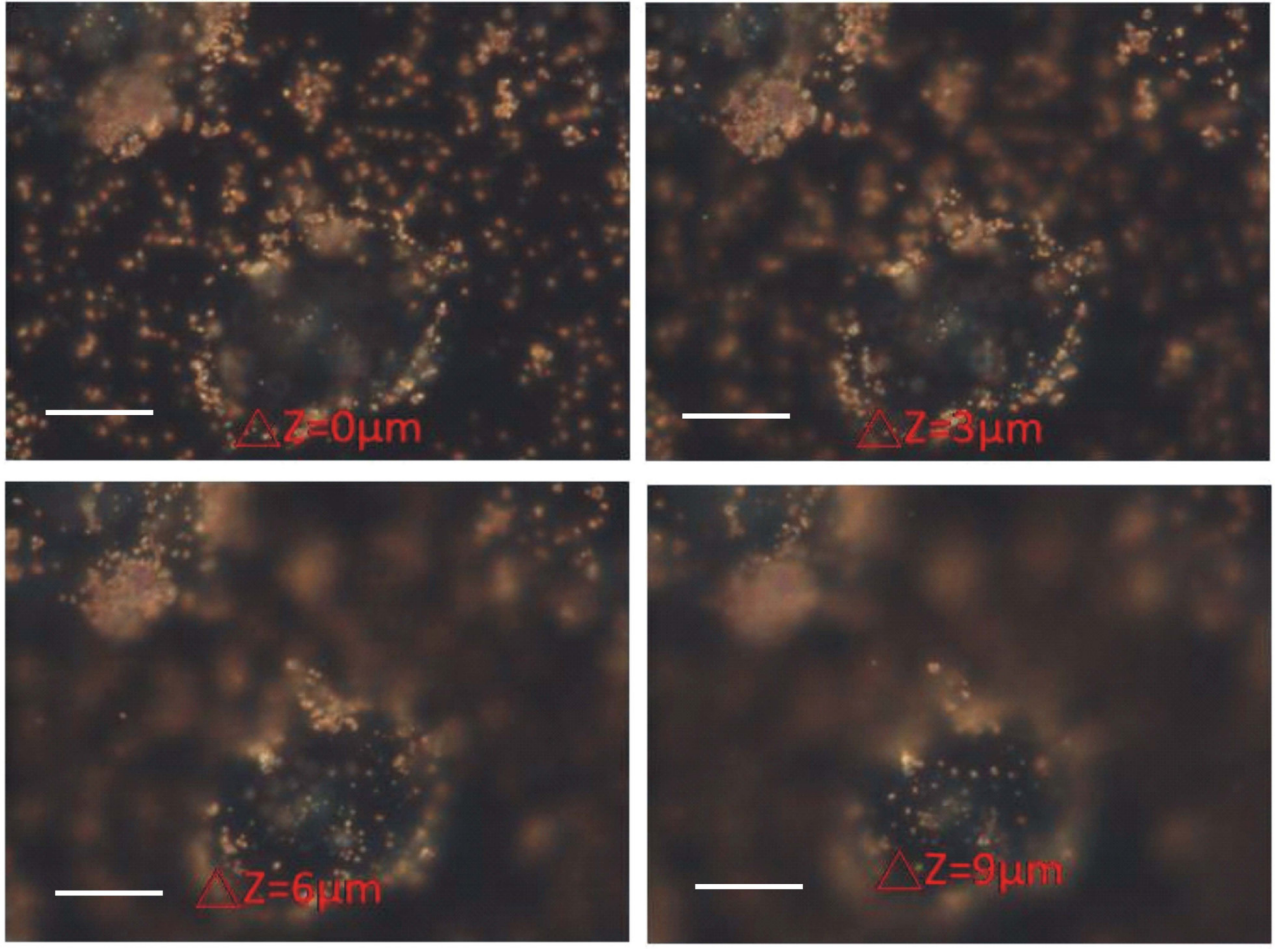
**The images for 110-nm-AuNPs at different heights of the cell**. The interaction time was two hours. The 110-nm-AuNPs show strong orange spots in the dark-field image. There were almost no vesicles found in the cells. Most of the 110-nm-AuNPs were on the cells. The scale bar is 10 μm.

We studied the interactions of AuNPs with two kinds of cancer cells, non-small lung cancer cells (CL1-0) and HeLa cells. Additional file 2 shows a movie for 70 nm AuNPs and CL1-0 cells for different interaction times. In this experiment, the 70 nm AuNPs were first injected into the cell chamber. After ten minutes, new culture medium was injected to the chamber to wash the unbounded AuNPs. The images were then recorded by the colour CCD with an exposure time of 100 ms. The interval between images was 5 minutes and the overall recording time was 1.5 hours. This movie shows the aptamer-modified AuNPs attached to the ECM and moving towards the cells. Few vesicles were found during this period. Most AuNPs were not taken up by the cells, but instead moved directly to the apical surfaces of the cells. To identify the positions of AuNPs, the sample was scanned over different cellular heights. Figure 3 shows the dark field CCD images at different times and heights of the cell. It confirmed that many AuNPs were attached to the apical surface without being internalised. Figure 4 shows the images for 110 nm AuNPs taken at different heights of the cell. The interaction time was two hours. The 110 nm AuNPs were visualised as strong orange spots in the dark field image. There were almost no vesicles found in the cells.

**Figure 5:**
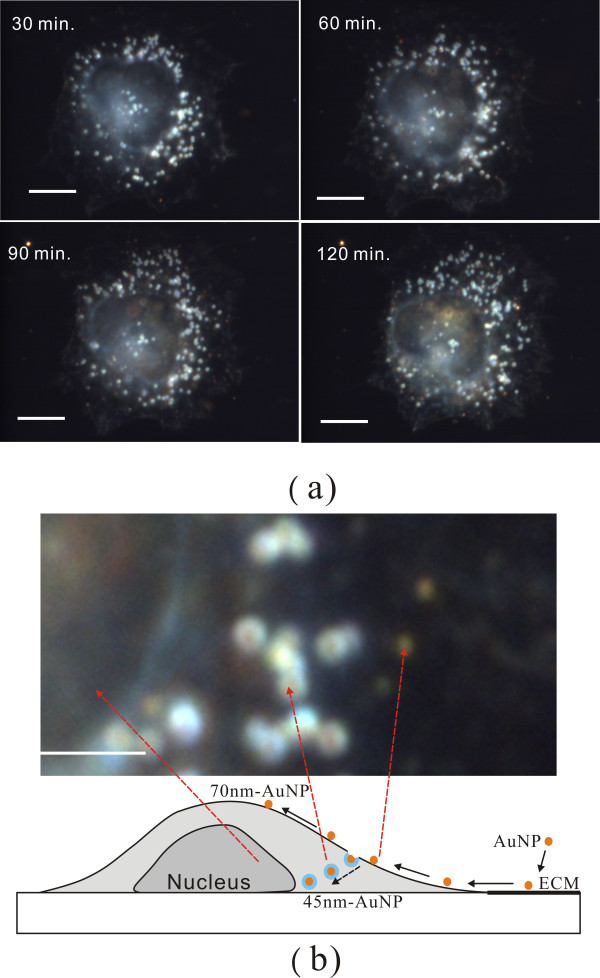
**The dark-field images for 45-nm-AuNPs and cells**. (a) The dark-field CCD images for 45-nm-AuNPs and a CL1-0 cell at different interaction time. The focus plane was fixed near the glass substrate, i.e. Δz = 0. The scale bar is 10 μm. (b) An enlarged image of Figure 5(a) for 120 minutes interaction time. The AuNPs showed yellow colors. When they were uptaken by the cell, the AuNPs became orange with blue surroundings. The picture demonstrated the movement and uptake of AuNPs for 45 nm and 70 nm AuNPs. Many 45-nm AuNPs are moved and then uptaken by cells, while many 75-nm-AuNPs only moved to the apical surface of the cells.

The behaviour of 45 nm AuNPs was quite different from 70 nm and 110 nm AuNPs. Additional file 3 shows a movie for 45-nm-AuNPs interacting with CL1-0 cells. The interval between images was 5 minutes and the overall recording time was 2 hours. This movie shows the aptamer-modified AuNPs attached to the ECM and moved towards the cells. However, it was found that many AuNPs did not attach to the apical surface of the cell. Instead, they entered the cells through endocytosis and accumulated in endocytic vesicles. Figure 5(a) shows the images at different interaction times. The numbers of vesicles containing AuNPs increased with time. Figure 5(b) shows the enlarged scattering image of 45 nm AuNPs and vesicles containing AuNPs. The AuNPs were visualised as yellow. When they were endocytosed, the AuNPs were visualized as orange surrounded with blue. From the above movies and images, the movement and uptake of 45 nm and 70 nm AuNPs are depicted in Figure 5(b). Many 45 nm AuNPs moved into the cells and then entered cells through endocytosis, while many 75 nm AuNPs only moved along the cell surface.

### 3D distribution of AuNPs

**Figure 6:**
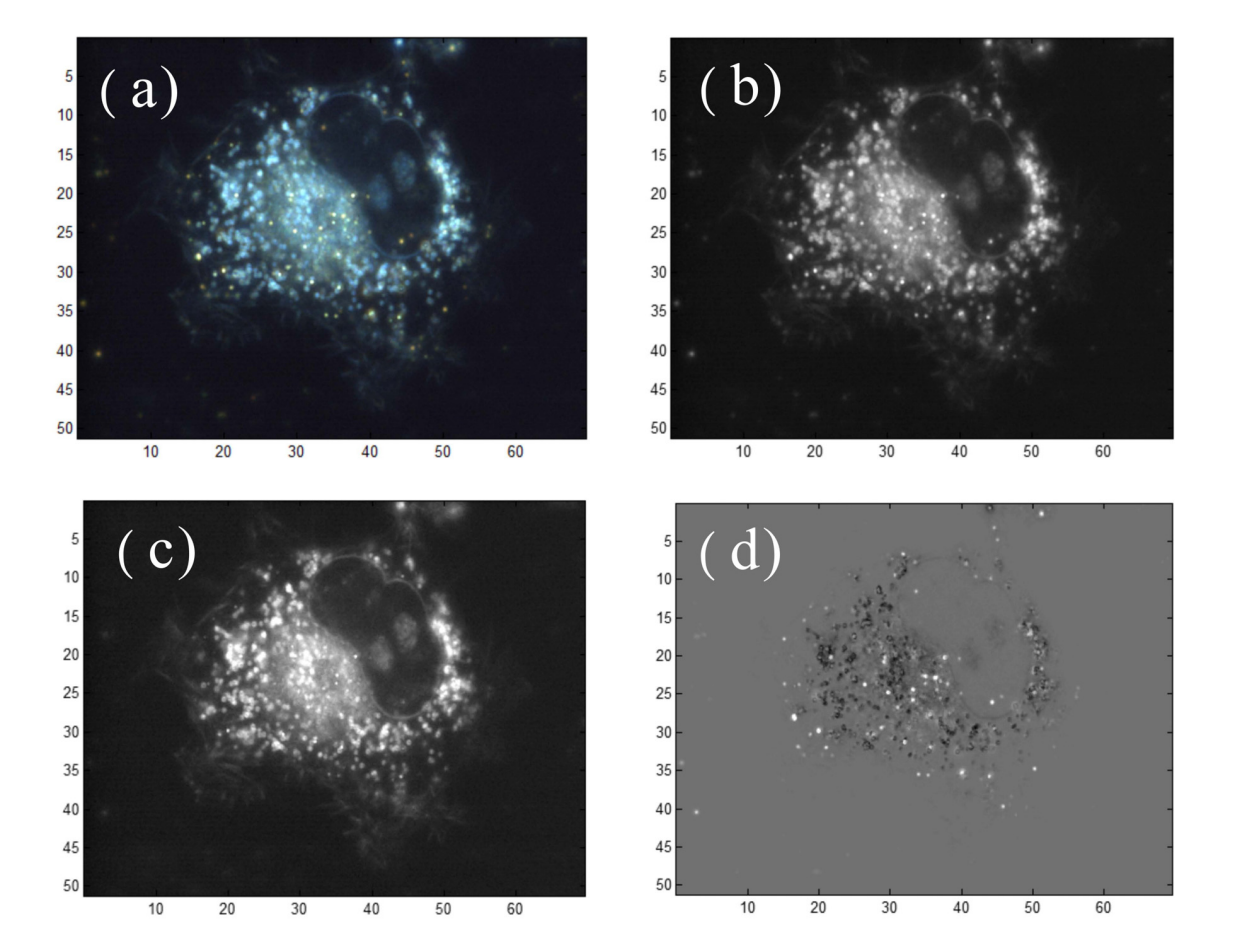
**The image process for enhancing the contrast of AuNPs**. (a) The dark-field optical image of a CL1-0 cell with 45-nm-AuNPs observed by a 60× oil lens and a color CCD. The AuNPs show orange colors in the dark-field image. There are white brighter regions appeared in the cell due to the scattering from large organelles. (b) The monochromatic image of the yellow component, (c) The monochromatic image of the blue component. Compared both images, the AuNPs have a higher contrast in the yellow image. The organelles is brighter in the blue image. (d) The combination of the Y and B images by using the (Y-B) calculations. The image process results in an image with bright AuNP spots and dark organelles. The unit is μm.

We found that different sizes of AuNPs interact with cells differently. The uptake rate can be quantitatively estimated by mapping 3D distributions of AuNPs. In these experiments, we used the difference of scattering spectra to distinguish AuNPs and cellular organelles. For example, Figure 6(a) shows a colourful scattering image. The cell was a CL1-0 cell and the AuNP size was 45 nm. A single AuNP shows as a yellow spot in the scattering image. There are other brighter regions in the cell due to the scattering of organelles. To exclude the organelles and identify the positions of AuNPs, we divided the image into the colours red (R), green (G) and blue (B). The (G+R)/2 image yielded a yellow image (Y) which had a stronger scattering intensity for AuNPs as seen in Figure 6(b). On the other hand, the organelles were brighter in the blue image (Figure 6(c)). If we applied the image process of (Y-B), the colour image then became a grey image which was positive for AuNPs and negative for organelles, as shown in Figure 6(d). AuNPs in this grey image were easily identified using computer-based image analysis.

**Figure 7:**
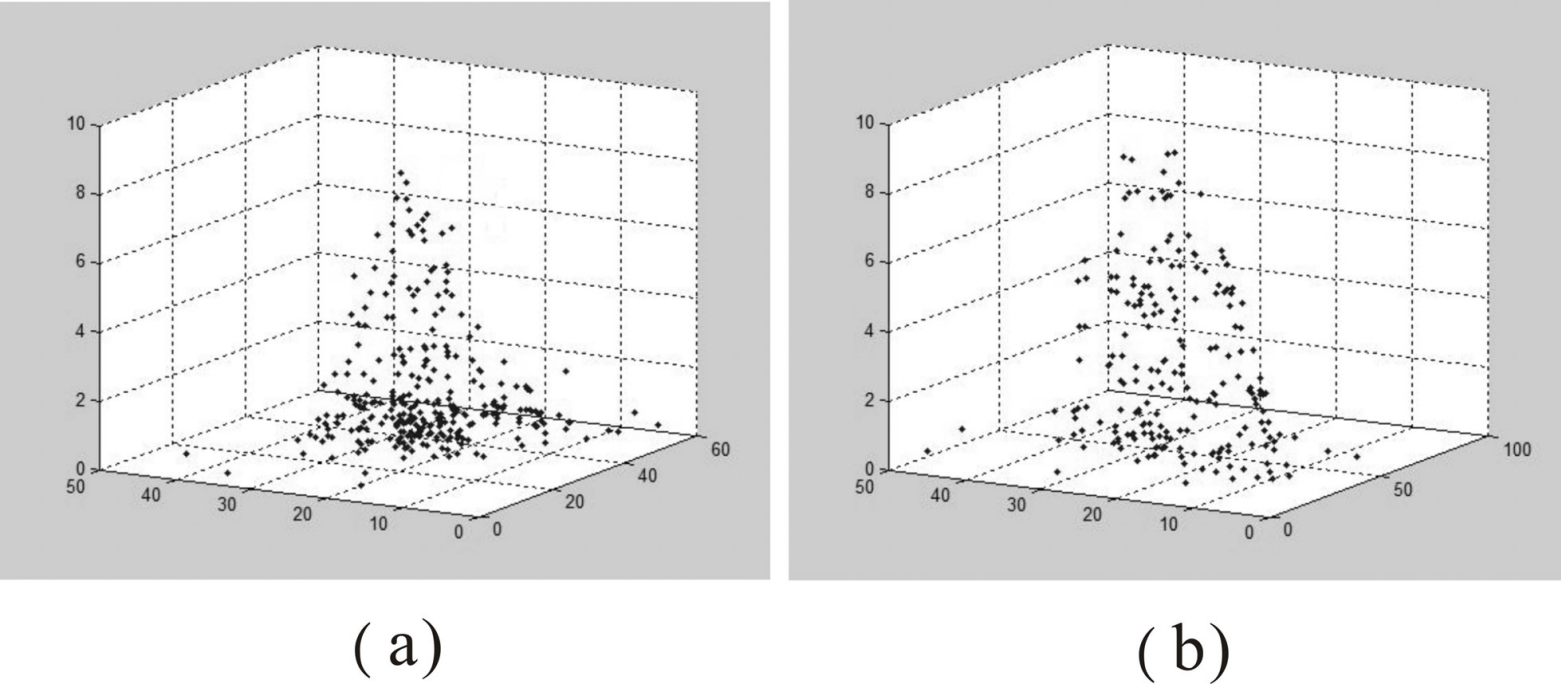
**The 3D images of the distribution of AuNPs**. (a) The calculated 3D image for 45-nm-AuNP aggregates distribution on a HeLa cell. (b) The calculated 3D image for 70-nm-AuNP aggregates distribution on a HeLa.

In this work, we developed a Matlab program to find the 3D positions of AuNPs in the cell. First, the colour images at different focal planes (z) were transformed into gray images by using the (Y-B) algorithm. Then the gray images for different focal planes were all projected onto the same x-y plane. In the processed images, only AuNPs show bright spots. The central position of every bright spot was recorded as the x-y position (xp, yp) of AuNPs. The z position (zp) for each AuNP was then determined by finding the maximum scattering intensity, *I*(xp, yp, zp) along the z -direction at a fixed (xp, yp) position. The computer program calculated the spot one by one. At last, all the (xp, yp, zp) points rendered the 3D distribution of AuNPs. Figure 7(a) shows the 3D distribution of 45 nm AuNPs in a CL1-0 cell. From this figure, it can be seen that many 45 nm AuNPs were located in the cytoplasm and at the bottom of the cell. Figure 7(b) shows the 3D distribution of 70 nm AuNPs in a CL1-0 cell. The image indicates that many 70 nm AuNPs were located on the cell surface. It indicates that morphology of the cell can be reconstructed by these large AuNPs.

### Quantitative calculation of the endocytosis

**Figure 8:**
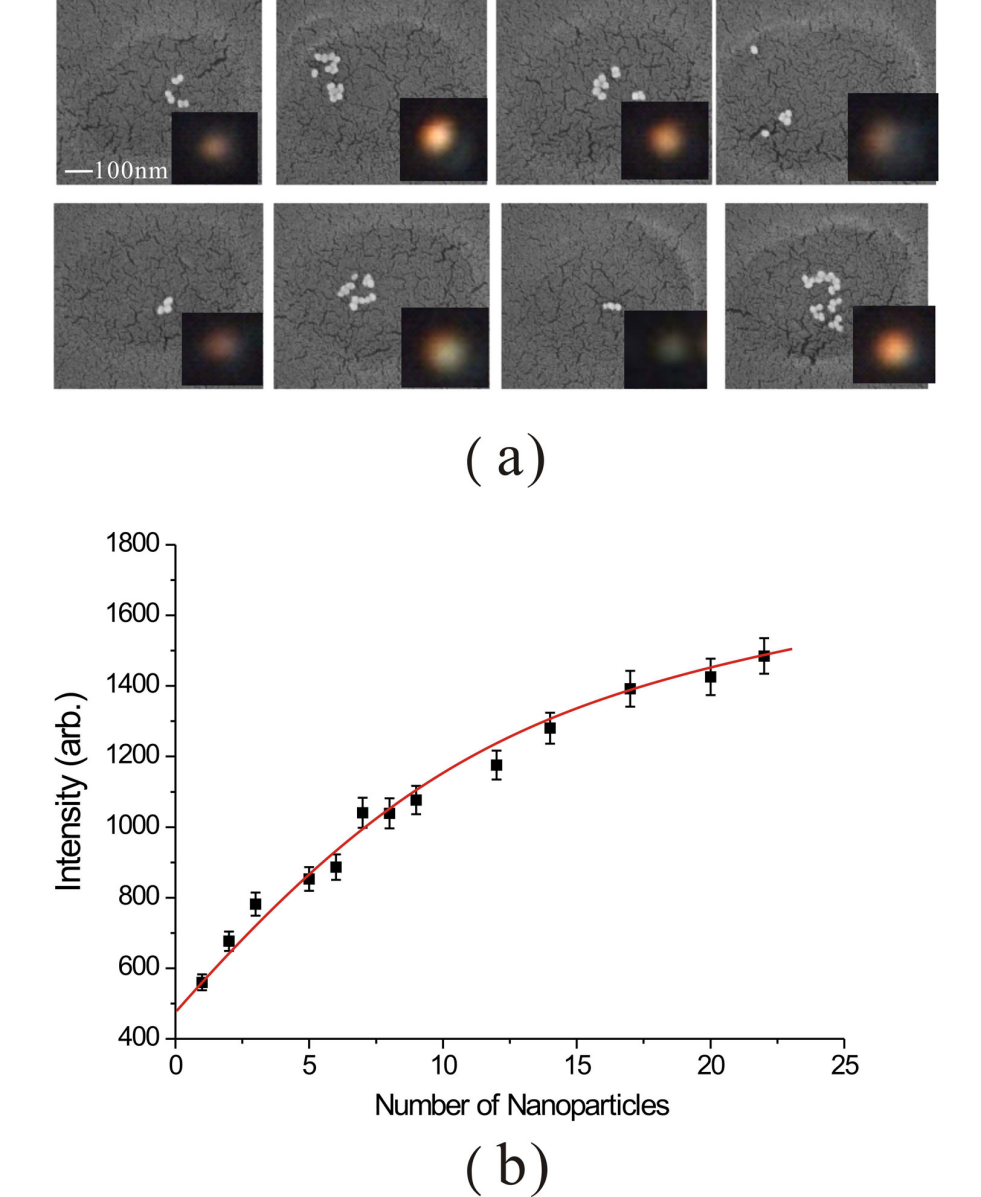
**The scattering intensity as a function of AuNP numbers**. (a) The measured optical scattering images and the corresponding SEM images for different aggregates of AuNPs. (b) The scattering intensities as a function of AuNP numbers. The scattering intensity was increased with the number of AuNPs.

To quantitatively calculate the uptake numbers of AuNPs, we have to know the AuNP number at each (xp, yp, zp) position. It is noted that previous endocytosis studies by electron microscope have shown that AuNPs mostly localise in vesicles (300 to 500 nm in size) and most of the AuNPs are in aggregated form [9]. Because the limited spatial resolution of the optical microscopy, the number of AuNPs in each aggregate cannot be simply identified by the optical images. Nevertheless, the scattering intensity *I*(xp, yp, zp) for each aggregate is different. The scattering intensity is increased with the AuNP number in the aggregate. To estimate the AuNP numbers in each aggregate, calibration curves between the scattering intensities and the corresponding AuNP numbers have to determined. In the experiment, we put different sizes of AuNPs in an array of 500-nm-holes. The scattering of AuNPs in the hole can be used to mimic the scattering of AuNP aggregate in the vesicle. The preparation of the samples were described in the Method section. Figure 8(a) shows the measured scattering images and the corresponding SEM images for different aggregates of 45-nm-AuNPs. We found that the scattering intensity was increased with the number of AuNPs. Using the curve presented in Figure 8(b) and the measured scattering intensity *I*(xp, yp, zp) for the AuNP aggregates, we can quantitatively estimate the AuNP numbers at each (xp, yp, zp) position.

**Figure 9:**
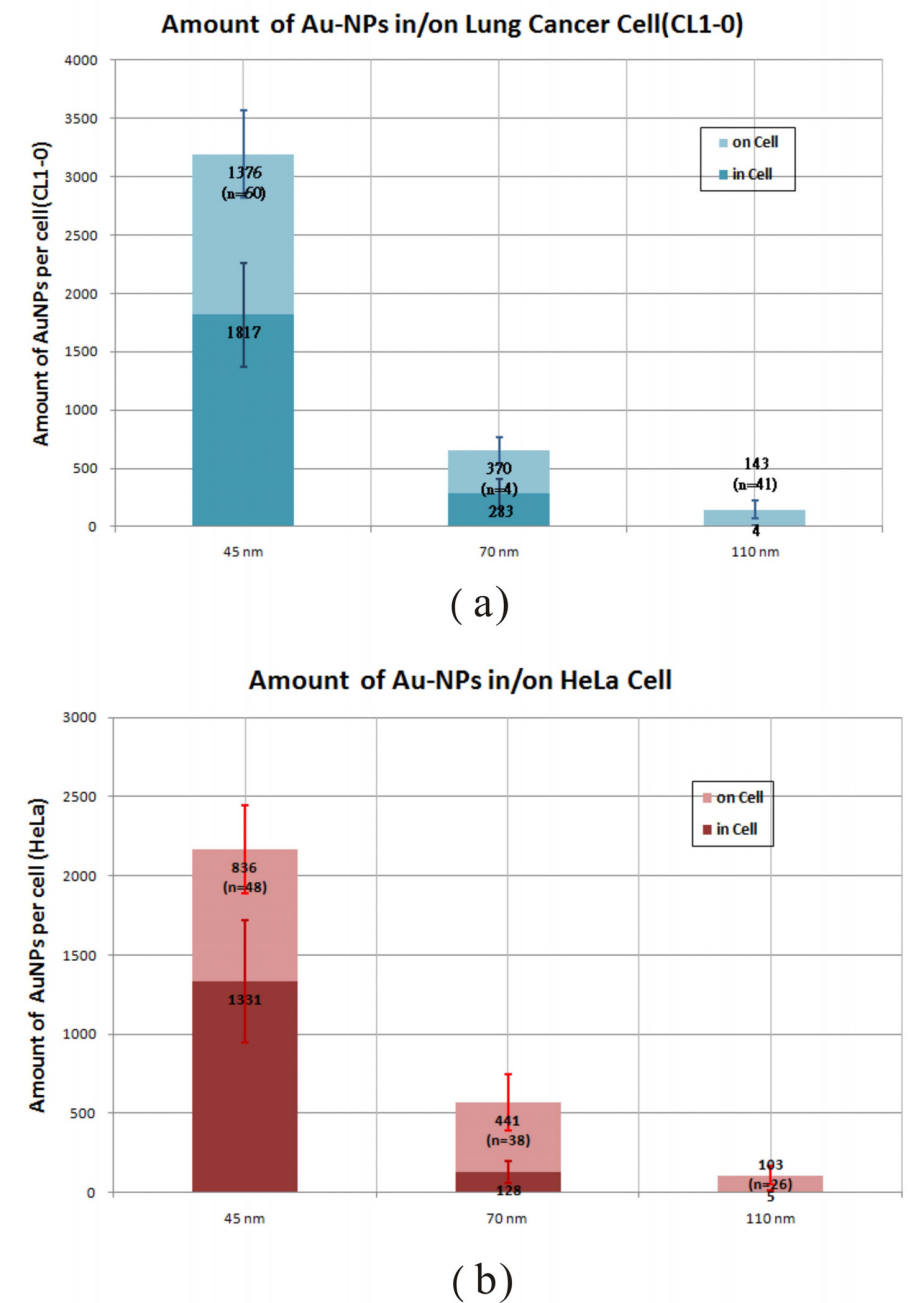
**The statistical results for the uptake of different AuNPs**. (a) CL1-0 cells and (b) HeLa cells for AuNPs of 45 nm, 70 nm and 110 nm diameters. The statistics show that the total amounts of AuNPs interacted with cells has a maximum value for 45-nm-AuNPs. The percentage of AuNPs in cells also has a maximum value for 45-nm-AuNPs. The *n *indicates the number of cells investigated.

The uptake rate of AuNPs was defined by the number of AuNPs in the cell divided by the total number of AuNPs. The total AuNP number was obtained by summing up AuNP numbers for all aggregates. The AuNP number on the cell was calculated by manually locating the AuNP positions (xp, yp, zp) on the cell from the 3D distribution as seen in Figure 7. The number on the cell was then calculated by summing up the AuNP numbers for the selected (xp, yp, zp) positions. The number of AuNPs in the cell was obtained by subtracting the AuNP number on the cell from the total AuNP number. Figure 9(a) shows the statistical results for CL1-0 cells using AuNPs 45 nm, 70 nm and 110 nm in diameter. The statistical results show that the total amount of AuNPs which interacted with cells had a maximum value for 45 nm AuNPs. The total amount is consistent with the result measured by using inductively coupled plasma atomic emission spectroscopy and transmission electron microscopy [11]. The amount for 45 nm AuNPs was about five times higher than for the 70 nm AuNPs. We used the 3D images to estimate the AuNPs in or on the cells. The percentage of AuNPs in cells also had a maximum value for the 45 nm AuNPs, demonstrating that about 58% of the AuNPs entered the cells. The cellular uptake was deceased for large AuNPs, to about 43% for 70 nm AuNPs and almost zero for 110 nm AuNPs. The same property of endocytosis was also found for HeLa cells, with the statistical results shown in Figure 9(b). The percentage of AuNPs in HeLa cells also had a maximum value for the 45 nm AuNPs, which was 61% inside the cells. It was about 23% for 70 nm AuNPs and almost zero for 110 nm AuNPs. These results verified that 45 nm AuNPs are better than larger-sized AuNPs for uptake into cancer cells.

## Discussion

The reason for size-dependent endocytosis of AuNPs can be explained by the thermodynamic model of the many-NP-cell system [24-26] for receptor-mediated endocytosis [27,28]. There are two kinds of competitive energy important for endocytosis of nanoparticles (NPs). One is the binding energy between ligands and receptors. This energy refers to the amount of ligand-receptor interaction and the diffusion kinetics for the recruitment of receptors to the binding site. The other is the thermodynamic driving force for wrapping. The thermodynamic driving force refers to the amount of free energy required to drive the NPs into the cell. These two factors determined how fast and how many NPs are taken up by the cell. For NPs with a diameter smaller than 40 nm, the docking of a single small NP will not produce enough free energy to completely wrap the NPs on the surface of the membrane. This could prevent the uptake of the single NP by endocytosis. For the smaller NPs to go in, they must be clustered together and thus take a long diffusion time. Therefore, the uptake amount is much smaller than 50 nm NPs. For NPs with a diameter larger than 80 nm, endocytosis rarely occurs. The depletion of free receptors limits the ligand-receptor binding energy for forming a large membrane curvature. Almost all NPs are only partially wrapped in the membrane. Between both regions, the optimal NP diameter has been identified at which the cellular uptake of NPs is maximised [29-31]. The optimal diameter for AuNPs falls in the range of 40-60 nm for reasonable values of membrane bending rigidity and ligand-receptor binding energy.

In the optical scattering study of AuNPs and cells, we investigated particle sizes from 45 nm to 110 nm. AuNPs can be prepared as small as 5 nm. However, it is hard to identify small nanoparticles in the cells simply by using scattering images. As indicated in Eq. 1, the scattering cross-section is greatly reduced when particle diameter is reduced. For AuNPs with a diameter smaller than about 30 nm, the scattering signal will be smaller than the micron-sized vesicles and is hard to be identified. Therefore, the proposed 3D scattering method is suited only for medium-sized AuNPs. With this particle size, the scattering signals of vesicles and AuNPs are comparable. It should be noted that these medium-sized AuNPs are of great interest than other sizes of AuNPs for endocytosis studies. Previous experiments using TEM and fluorescence microscopy have all indicated that AuNPs with a size of 40-60 nm have the best cellular uptake efficiency [7-10]. For small and large AuNPs, most remain bound to the membrane. Hence, for drug delivery by AuNPs, this proposed method is very useful for long-term tracking of the process of endocytosis without any labelling.

## Conclusions

We studied endocytosis of AuNPs with different sizes (45 nm, 70 nm and 110 nm) in various cells (the human cancer cell lines, CL1-0 and HeLa). Compared with previous methods using transmission electron microscopy and fluorescence microscopy, the proposed method provides a simple way to define whether AuNPs are in the cytoplasm or adhered to the membrane of living cells. Using the spectroscopic difference between AuNPs and cell organelles, a colour CCD with a simple post-process can easily identify the positions of AuNPs. From the 3D distributions of AuNPs, we have experimentally confirmed that endocytosis of AuNPs is size dependent. For the cells we studied, the optimal size for the uptake into cells was around 45 nm. These results suggest that a particle size of 45 nm has the highest efficiency for drug delivery by AuNPs. On the other hand, large AuNPs which remain bound to the cell membrane can be used to reconstruct the morphology of the cell.

## Competing interests

The authors declare that they have no competing interests.

## Authors' contributions

Conceived and designed the experiments: PKW SHW. Performed the experiments: SHW CWL. Analyzed the data: SHW PKW. Contributed reagents/materials/analysis tools: CWL AC. Wrote the paper: PKW. All authors read and approved the final manuscript.

## Supplementary Material

Additional file 1 **The SEM images for different sizes of AuNPs**. (a) 13 nm, (b) 45 nm, (c) 70 nm and (d) 110 nm AuNPs on glass substrates. Click here for file

Additional file 2 **The movie for 70-nm-AuNPs and a HeLa cell**. The images were observed by using the dark-field microscope. The CCD exposure time was 100 ms, the interval between images was 5 minutes and the overall recording time was 1.5 hours. Click here for file

Additional file 3 **The movie for 45-nm-AuNPs and a CL1-0 cell**. The images were observed by using the dark-field microscope. The CCD exposure time was 100 ms, the interval between images was 5 minutes and the overall recording time was 2 hours. Click here for file
