# Supplementary figures and images for: Size-dependent endocytosis of gold nanoparticles studied by three-dimensional mapping of plasmonic scattering images
Source: J Nanobiotechnology. 2010 Dec 20;8:33. doi: 10.1186/1477-3155-8-33 (PMC3236302; doi:10.1186/1477-3155-8-33)

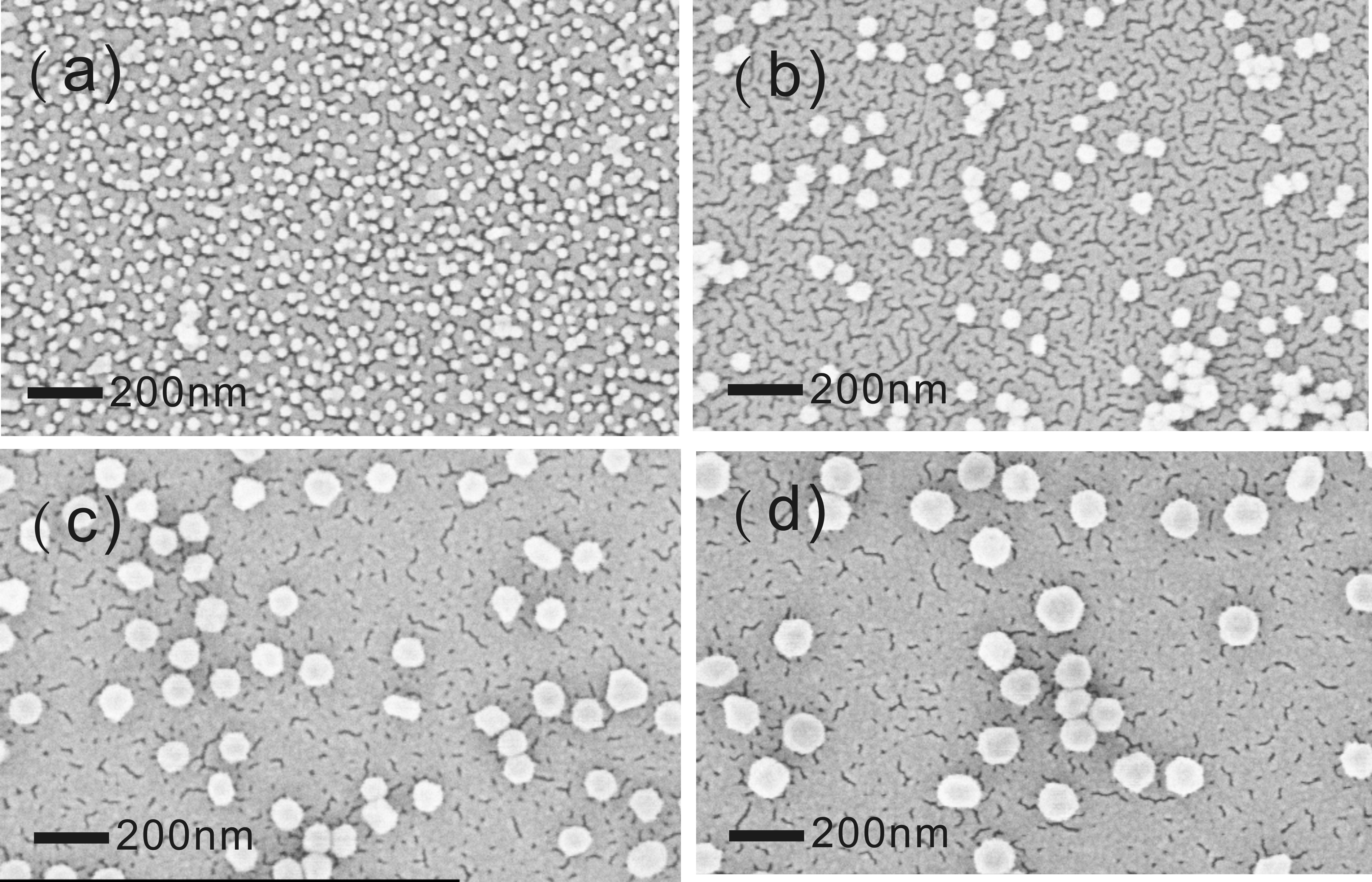

Supplement: Additional file 1 — The SEM images for different sizes of AuNPs. (a) 13 nm, (b) 45 nm, (c) 70 nm and (d) 110 nm AuNPs on glass substrates. [file 1477-3155-8-33-S1.TIFF]
